# Rnf40 Exacerbates Hypertension‐Induced Cerebrovascular Endothelial Barrier Dysfunction by Ubiquitination and Degradation of Parkin

**DOI:** 10.1111/cns.70210

**Published:** 2025-01-08

**Authors:** Chengkun Kou, Xu Zhao, Xin Fan, Runmin Sun, Wenting Wang, Miaomiao Qi, Lulu Zhu, Xin Lin, Jing Yu

**Affiliations:** ^1^ Hypertension Center, The Second Hospital & Clinical Medical School Lanzhou University Lanzhou Gansu China; ^2^ Cuiying Biomedical Research Center, The Second Hospital & Clinical Medical School Lanzhou University Lanzhou Gansu China

**Keywords:** cerebrovascular endothelial barrier, cognition, endothelial cell, hypertension, mitophagy, Rnf40

## Abstract

**Aims:**

We aimed to investigate the role of Rnf40 in hypertension‐induced cerebrovascular endothelial barrier dysfunction and cognitive impairment.

**Methods:**

We employed microarray data analysis and integrated bioinformatics databases to identify a novel E3 ligase, Rnf40, that targets Parkin. To understand the role of RNF40 in hypertension‐induced cerebrovascular endothelial cell damage, we used pAAV‐hFLT1‐MCS‐EGFP‐3×Flag‐mir30shRnf40 to establish an Rnf40‐deficient model in spontaneously hypertensive rats (SHRs). We also evaluated the cerebrovascular endothelial barrier function, cerebral blood flow, and cognitive performance.

**Results:**

We observed reduced mitophagy in cerebrovascular endothelial cells of SHRs compared with that in Wistar‐Kyoto rats. Rnf40 facilitated K48‐linked polyubiquitination and degradation of Parkin, thereby inhibiting mitophagy. In the Rnf40‐deficient SHR model, knocking down Rnf40 restored mitophagy in cerebrovascular endothelial cells. Additionally, levels of tight junction proteins and cerebrovascular endothelial barrier function improved following Rnf40 downregulation. Rnf40 depletion also improved global cognitive performance and restored cerebral blood flow in SHRs.

**Conclusion:**

Our findings suggest that increased Rnf40 levels exacerbate hypertension‐induced cerebrovascular endothelial barrier dysfunction by ubiquitinating Parkin.

## Graphical abstract

Schematic diagram of the mechanism that Rnf40 exacerbates hypertension‐induced cerebrovascular endothelial barrier dysfunction (Created in BioRender. Kou, C. (2024) BioRender.com/v22o557).

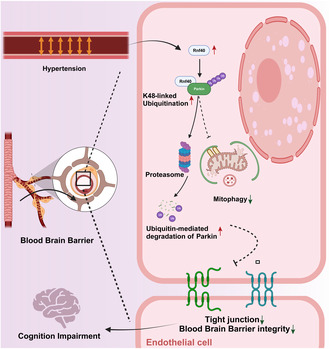

## 1 Introduction

Hypertension is a significant global public health issue, affecting an estimated 1.4 billion people worldwide, with effective management observed only in 14% of these cases [1]. Hypertension can reportedly damage various organs [2], particularly the brain. Cerebrovascular impairment induced by hypertension significantly contributes to cognitive dysfunction and vascular dementia [3, 4]. Additionally, high blood pressure directly impacts cerebrovascular endothelial cells, causing vasoregulatory and barrier dysfunctions [5]. Circulating angiotensin II (Ang II) reportedly disrupts the blood–brain barrier (BBB), which consequently increases permeability and downregulates tight junction proteins in spontaneously hypertensive rats (SHRs) [6]. A retrospective study also reported heightened BBB permeability among patients with hypertension [7]. However, research on the effects of hypertension on the endothelial barrier function remains limited, and the underlying mechanisms are not fully understood.

Mitophagy, a selective autophagic process, eliminates and recycles dysfunctional mitochondria, preserving mitochondrial function and quality [8]. This process interacts with other biological mechanisms and impacts overall health [9]. Recent research highlights the role of mitophagy in cardiovascular diseases [10], neuroprotection [11], and skeletal health [12]. It also maintains microvascular endothelial function. In cardiac microvascular ischemia–reperfusion (IR), activating the mitophagy pathway enhances the endothelial barrier function and integrity [13]. However, mitophagy is inhibited in IR, and restoring it by targeting neutrophil extracellular traps effectively reduces endothelial cell impairment [14]. The PTEN‐induced putative kinase 1/Parkin pathway, critical for mitophagy, is extensively studied. Parkin, an E3 ubiquitin ligase encoded by *PARK2*, recruits ubiquitin to bind substrate proteins, which are recognized by the proteasome for degradation [15]. Regulating Parkin expression reportedly effectively controls mitophagy [16]; therefore, targeting Parkin to regulate mitophagy offers a promising strategy for treating hypertension‐induced cerebrovascular endothelial barrier dysfunction.

The ubiquitin–proteasome system (UPS) governs a wide range of biological reactions, including proteasome‐mediated protein degradation. UPS plays a vital role in hypertension and hypertension‐related organ damage [17, 18]. The E3 ubiquitin ligase RING finger protein (Rnf40) monoubiquitylates histone H2B at lysine 120.17 Rnf40 reportedly targets Syntaxin 1 for degradation, potentially affecting learning and memory [19]. Moreover, Rnf40 plays a crucial role in the pathogenesis of multiple diseases, including inflammatory bowel disease [20], cancers [21, 22], and cancer‐associated osteolysis [23], highlighting its potential as a therapeutic target.

To address these gaps in the literature, in this study, we aimed to investigate the role of Rnf40 in hypertension‐induced cerebrovascular endothelial barrier dysfunction and cognitive performance. Specifically, we examined mitophagy deficiencies in the cerebrovascular endothelial cells of SHRs and aimed to elucidate the mechanisms by which Rnf40 influences Parkin ubiquitination and degradation, as well as its impact on mitophagy. We further knocked down Rnf40 in cerebrovascular endothelial cells of SHRs and monitored its impact on proteasome system cerebrovascular endothelial barrier function and cognitive performance. Collectively, we believe that our findings would help identify a potential therapeutic target for hypertension‐induced cognitive impairment.

## 2 Methods

### 2.1 Animals

This experiment was approved by the Institutional Animal Care and Use Committee of Lanzhou University Second Hospital (NO. 2023‐037) and adhered to the ARRIVE guidelines. Specific pathogen‐free male Wistar‐Kyoto (WKY) rats and SHRs, aged 12 weeks, were procured from Vital River Laboratory Animal Technology Co. Ltd. (Beijing, China). All rats were housed in the Lanzhou University Animal Laboratory Center under standard laboratory conditions with free access to food and water, maintained at a stable temperature of 25°C, 65%–70% humidity, and a 12–12‐h light–dark cycle.

For the Rnf40 knockdown models, the pAAV‐hFLT1‐MCS‐EGFP‐3 × Flag‐mir30shRnf40 vector (OBiO Techn Co. Ltd., China) was administered intravenously to SHRs (100 μL of sterile saline containing 1 × 10^13^ viral genome copies) via the tail vein under isoflurane anesthesia (4% for induction and 2% for maintenance) at 30 weeks old. The rats were subsequently housed for 4 weeks of fed under normal conditions. TRV027 (0.5 μg/kg/min) was used as a positive control to reduce blood pressure, administered via osmotic mini pumps (Cat No. 2001D, Alzet Corporation, California) for 28 days beginning at 30 weeks of age, as previously described (Figure 6A). In line with prior research, blood pressure measurements were recorded before and after the intervention using a tail‐cuff plethysmography system (IITC Life Science MRBP System, Woodland Hills, USA) [24, 25].

### 2.2 Cell Lines and Culture

The human cerebral microvascular endothelial cell line (hCMEC/d3) was purchased from Hunan Fenghui Biotechnology Co. Ltd. (Cat No. CL0530), and the HEK‐293T cell line was obtained from Wuhan Servicebio Technology Co. Ltd. (Cat No. STCC10301P). Both cell lines were cultured in Dulbecco's modified Eagle's medium (DMEM), supplemented with 10% fetal bovine serum (FBS), 100 mg/mL of penicillin, and 100 mg/mL of streptomycin at 37°C in a humidified chamber with 5% CO_2_.

### 2.3 Co‐Immunoprecipitation Assay

HEK‐293 T cells were transfected with Flag‐Parkin/Flag‐GFP and His‐Rnf40 for 24 h. Subsequently, cells were treated with 20 μM MG‐132 for 4 h, lysed, and immunoprecipitated using the Flag M2 Affinity Gel (Cat No. A2220, Merck, Germany), followed by Western blotting with anti‐His antibodies.

### 2.4 Microscale Thermophoresis Assay

As detailed in previous studies, the microscale thermophoresis assay (MST) quantified the interaction between Rnf40 and Parkin [26, 27]. We performed the MST assay using a Monolith NT.115 system (NanoTemper Technologies, Munich, Germany). Parkin was labeled using the manufacturer's kits (MonolithTM RED‐NHS, NanoTemper Technologies, Germany). Parkin solutions were prepared in 130 mM NaHCO_3_ (8.3) with 50 mM NaCl, and Rnf40 solutions were prepared in 10 mM phosphate‐buffered saline (PBS) (pH 7.4). Samples were added to Monolith capillaries (MO L022, NanoTemper Technologies, Germany) and analyzed using MST.

### 2.5 In Vivo Ubiquitination Assay

HEK‐293T cells were transfected with Flag‐Parkin, His‐Rnf40, and HA‐Ub for 24 h. Subsequently, the cells were treated with 20 μM MG‐132 for 4 h, lysed, and immunoprecipitated using Flag M2 Affinity Gel (Cat No. A2220, Merck, Germany) followed by Western blotting using anti‐HA antibodies to detect the levels of Parkin ubiquitination.

### 2.6 In Vivo and In Vitro Fluorescein Isothiocyanate (FITC)–Dextran Permeability Assay

The in vivo FITC–dextran permeability assay was conducted according to previously described protocols [28, 29]. In our study, we used 10‐kDa FITC–dextran (Cat No. M21464, AbMole, U.S.) to evaluate the BBB permeability in SHRs. FITC–dextran was suspended in PBS at a concentration of 3 mg/mL. Following anesthesia, 0.2 mL per 100 g body weight of FITC–dextran was administered through the jugular vein. After 10 min of circulation, the rats were perfused with PBS at 20 mL/min for 5 min. The relative fluorescence units (RFUs) of FITC–dextran in the cortex were quantified using a spectral scanning multimode reader (Varioskan Flash 5250030, Thermo Fisher, U.S.) at 520 nm. The permeability index was calculated as (cortex RFUs/cortex weight)/(serum RFUs/serum weight).

An in vitro FITC–dextran permeability assay was also performed. hCMEC/D3 cells were seeded in the upper chamber of a Transwell at a density of 5 × 10^5^ cells/cm^2^ and cultured continuously for 7 days to achieve confluence and establish a monolayer. Before the assay, 1 mg/mL of FITC–dextran was added to the upper chamber and incubated at 37°C for 10 min. The culture medium from the lower chamber was collected, and the Transwell insert was moved to a new well and incubated for an additional 10 min at 37°C. This process was repeated five times. The cumulative concentration of FITC–dextran in the collected medium was measured to assess permeability (Figure 4G).

The permeability coefficient (Papp) was calculated using the formula:

$$\text{Papp}=\mathrm{dQ}/\mathrm{dt}\;\left(1/A\cdot C0\right)$$

where *dQ*/*dt* represents the permeability rate, *A* is the cross‐sectional area, and *C*0 is the initial concentration of the donor solution [30].

### 2.7 Molecular Docking

Protein–protein docking simulations were performed to generate well‐fitted conformations of Rnf40 (PDB Code: 8GUI) within the potential binding pockets of Parkin (Alphafold‐predicted structure). Structures devoid of heteroatoms were utilized for docking via MOE. The entire Parkin protein was employed as the receptor, while the entire Rnf40 protein served as the ligand. Retained molecules with conformations exhibiting the lowest binding energies underwent analysis through 3D protein–ligand interactions.

### 2.8 Mitochondrial Membrane Potential

The mitochondrial membrane potential of hCMEC/d3 cells was evaluated using JC‐1 staining solution (M8650, Solarbio Life Science, China). After removing the culture medium, cells were incubated in a dark environment at 37°C for 20 min. Following incubation, cells were washed twice with staining buffer to remove excess stain before fresh medium was added. The samples were then analyzed to assess mitochondrial membrane potential via fluorescence microscope. Healthy cells with high mitochondrial membrane potential will have JC‐1 aggregates, which fluoresce red (excited at 550 nm), whereas cells with low mitochondrial membrane potential will predominantly have JC‐1 monomers, which fluoresce green (excited at 460 nm).

### 2.9 Mito‐SOX

Intracellular mitochondrial reactive oxygen species levels were measured using the mito‐SOX Red Mitochondrial Superoxide Indicator (HY‐D1055, MCE, China). After treating macrophages with diluted mito‐SOX for 10 min at 37°C, the cells were washed three times with 1× PBS. The fluorescence intensity was subsequently detected using a fluorescence microscope (excited at 550 nm).

### 2.10 Mitophagy Assays

A lentiviral vector containing pLV3‐CMV‐mito‐mKeima‐Puro was used to assess mitophagy statues in endothelial cells. The mKeima probe was detected using the confocal microscopy. This probe emits green fluorescence under neutral mitochondrial conditions (excited at 458 nm), while it emits red fluorescence when mitochondria undergo mitophagy in an acidic lysosomal environment (excited at 561 nm). The progression of mitophagy was analyzed by calculating the ratio of red to green fluorescence intensity using ImageJ software, providing insights into the dynamics of mitochondrial turnover.

### 2.11 Morris Water Maze

The Morris water maze (MWM) was used to assess memory deficits and spatial learning in rats. Rat activity was tracked using a video‐based system (XR‐XM101, XinRuan, China). The pool contained opaque water prepared by adding a washable white paint. A circular platform (diameter = 17 cm) was submerged 1 cm below the water surface in the midpoint of the fourth quadrant. The training lasted 4 days, during which rats underwent four 120‐s trials daily to locate the platform. The results of the trials were averaged. On the fifth day, the probe test was conducted by removing the platform, allowing rats to swim for 60 s while the time spent in each quadrant was recorded.

### 2.12 Statistical Analysis

All statistical analyses were performed using GraphPad Prism 9.0 (GraphPad Software Inc., San Diego, CA, USA) and R 4.3.2 (R Foundation for Statistical Computing, Vienna, Austria). The Shapiro–Wilk test was performed to assess normality of the data. Homogeneity of variance was assessed by Levene's test. Data are presented as mean ± standard deviation (SD). For normally distributed data, differences between groups were analyzed using either Student's *t*‐test for comparisons between two groups or a one‐way analysis of variance (ANOVA) with Tukey's post hoc test for multiple comparisons when more than two groups were involved. For data that did not exhibit a normal distribution, non‐parametric tests, including the Mann–Whitney *U* test (for two groups) or the Kruskal–Wallis test with Dunn's post hoc test (for more than two groups), were used. Statistical significance was defined as *p* < 0.05.

## 3 Results

### 3.1 Cerebrovascular Endothelial Barrier and Mitophagy Deficiency in SHRs

SHRs exhibit behavioral and neuropathological characteristics similar to those of patients in the early stages of cerebral small vessel disease (CSVD). BBB leakage becomes evident in SHRs at approximately 34 weeks [31]. In this study, we selected aged SHRs (34 weeks) to investigate mechanisms underlying the hypertension‐induced cerebrovascular endothelial barrier dysfunction and cognitive impairment.

**FIGURE 1:**
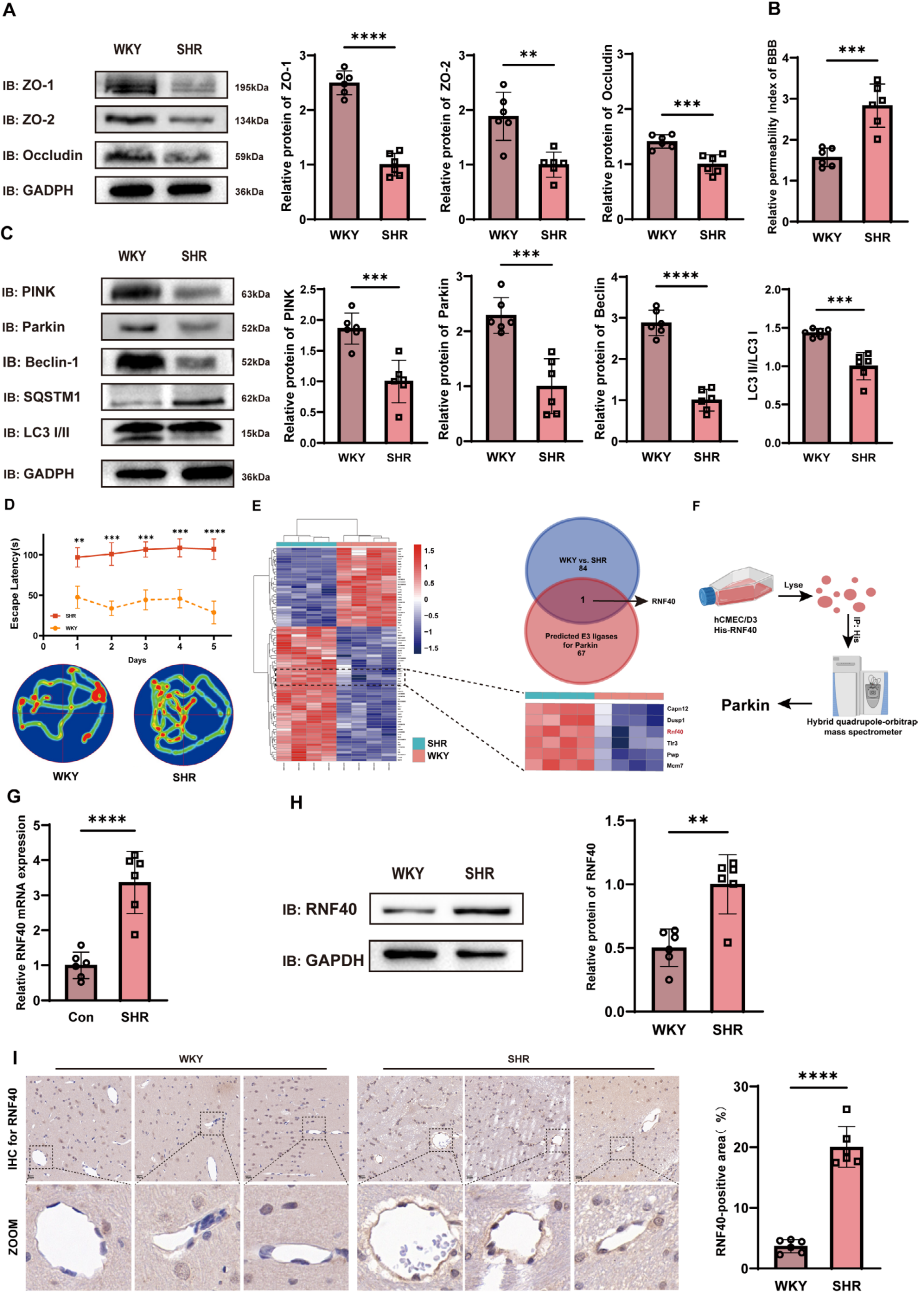
Deficiency in cerebrovascular endothelial barrier and mitophagy in SHRs, accompanied by Rnf40 overexpression. (A) Protein levels of tight junction proteins detected using Western blotting (*n* = 6). (B) Relative permeability index of the blood–brain barrier assessed using the FITC–dextran permeability assay. Statistical significance was determined using Student's *t*‐test (mean ± SD, *n* = 6). (C) Protein levels of mitophagy markers detected via Western blotting (mean ± SD, *n* = 6). (D) Escape latency and activity heatmap in the Morris Water Maze. Statistical analysis using Student's *t*‐test (mean ± SD, *n* = 8). (E) Differential gene expression between WKY rats and SHRs, with the Venn diagram identifying Rnf40 as a potentially overexpressed E3 ligase of Parkin in SHRs. (F) Mass spectrometry results depicting proteins interacting with Rnf40. (G) Relative Rnf40 mRNA expression in cerebrovascular endothelial cells of SHRs compared with that in WKYs via qRT‐PCR (mean ± SD, *n* = 6). (H) Representative immunoblot showing decreased tight junction protein levels in cerebrovascular endothelial cells of SHRs compared with that in WKYs (mean ± SD, *n* = 6). (I) Representative images from immunohistochemistry showing Rnf40 expression, with quantification of positive area percentage. Scale bar = 40 μm. Statistical analysis using Student's *t*‐test (mean ± SD, *n* = 6). SHRs, spontaneously hypertensive rats; WKY rats, Wistar‐Kyoto rats. **: *p* < 0.01; ***: *p* < 0.001; ****: *p* < 0.0001.

We confirmed via the tail‐cuff blood pressure monitoring that blood pressure levels of SHRs significantly exceeded those of WKY rats (Figure S1). To investigate cerebrovascular endothelial dysfunction induced by hypertension, we isolated brain vasculature according to previously published methods [32]. Subsequently, we analyzed the relative levels of endothelial cell biomarkers to confirm the enrichment of vascular elements. Western blotting data indicated that endothelial cell biomarker levels in the isolated vasculature significantly exceeded those in brain homogenate or supernatant (Figure S2A), ensuring the purity of vascular elements in this study. Using isolated vasculature, we compared tight junction protein levels between WKY rats and SHRs. We observed decreased levels in SHRs (Figure 1A); consistently, the FITC–dextran permeability assay revealed a significant increase in BBB permeability in SHRs (Figure 1B). Mitophagy protein levels were also decreased in these rats (Figure 1C). These results indicate tight junction dysfunction and mitophagy deficiency as characteristic features of cerebrovascular endothelial cells in SHRs.

Cognitive assessment using the MWM revealed that SHRs displayed a significantly increased escape latency compared with that in WKY rats (Figure 1D). In probe trials, SHRs spent significantly less time in the target quadrant than that by WKY rats (Figure S2B). Furthermore, we assessed brain structures and relative cerebral blood flow (rCBF) in SHRs using magnetic resonance imaging (MRI). No significant differences in cortical or hippocampal volumes were observed between SHRs and WKY rats (Figure S2D,E). However, arterial spin labeling (ASL) data showed significantly reduced rCBF in the cortex and hippocampus of SHRs (Figure S2F,G). Collectively, our data indicate cognitive impairment and reduced rCBF in the cortex and hippocampus of SHRs.

### 3.2 Rnf40 Overexpression in SHRs

To identify a key regulator of mitophagy deficiency in SHRs, we analyzed the GSE74288 dataset and identified 83 differentially expressed genes between WKY rats and SHRs (Figure 1E). Given the close association between Parkin ubiquitination and various diseases [33], we combined microarray data analysis with bioinformatics databases, identifying the E3 ligase Rnf40, which is overexpressed in SHRs and potentially an E3 ligase of Parkin (Figure 1E and Figure S2H,I). Using mass spectrometry (MS), we identified 202 proteins interacting with Rnf40, including Parkin (Figure 1F). Additionally, the relative Rnf40 mRNA expression was elevated in SHRs (Figure 1G). Western blot and immunohistochemistry results confirmed significantly increased Rnf40 protein levels in SHRs (Figure 1H,I). These findings suggest that Rnf40 interacts with Parkin and is overexpressed in SHRs.

### 3.3 Rnf40 Promotes Ubiquitination and Degradation of Parkin

**FIGURE 2:**
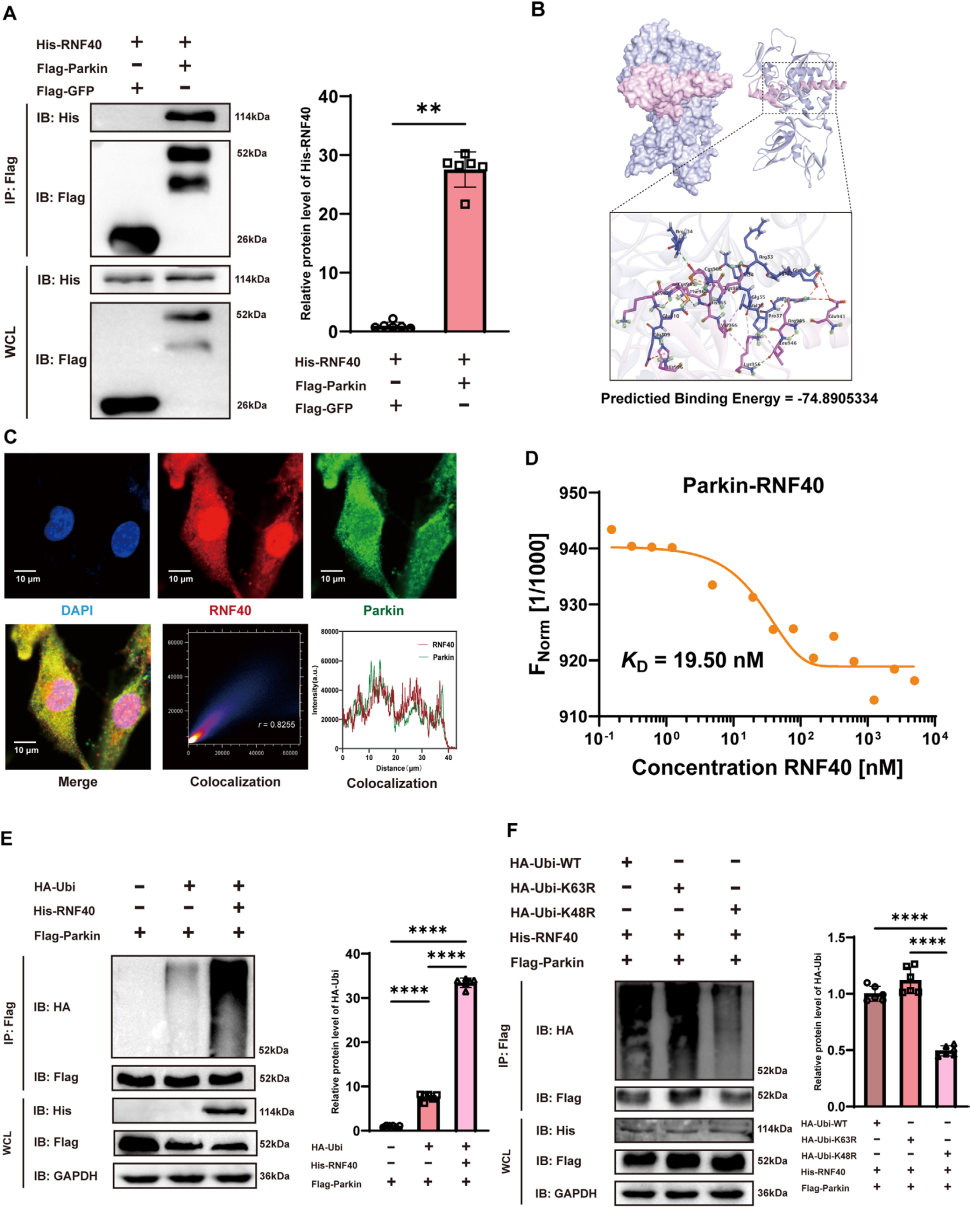
Rnf40 interacts with and ubiquitinates Parkin. (A) Detection of His‐Rnf40 protein in an anti‐Flag immunoprecipitate from HEK293T cells overexpressing Flag‐Parkin and His‐Rnf40 using Western blotting (mean ± SD, *n* = 6). (B) Structural overview and detailed 3D protein–ligand interaction analysis of Parkin and Rnf40. A salt bridge and over 10 hydrogen bonds exist between the two proteins. (C) Co‐localization analysis for Rnf40 and Parkin. The Pearson correlation coefficient was calculated. Scale bar = 40 μm. (D) Microscale thermophoresis assay depicting the binding of fluorescently labeled Parkin to Rnf40. Rnf40 titration from 0.15 nM to 5 μM yielded a KD of 19.5 nM. (E) HEK293T cells co‐transfected with Flag‐Parkin, HA‐Ub, and His‐Rnf40, followed by MG‐132 treatment (20 μM, 4 h). Flag‐Parkin was immunoprecipitated with an anti‐Flag antibody, and Parkin ubiquitination was detected with an anti‐HA antibody using Western blotting (mean ± SD, *n* = 6). (F) HEK‐293T cells transiently transfected with Flag‐Parkin, His‐Rnf40, and HA‐tagged wild‐type or mutant ubiquitin, then treated with MG‐132 (20 μM, 4 h). Flag‐Parkin immunoprecipitation using an anti‐Flag antibody reveals Parkin ubiquitination with an anti‐HA antibody using Western blotting (mean ± SD, *n* = 6). **: *p* < 0.01; ****: *p* < 0.0001.

To clarify the interaction between Rnf40 and Parkin, we detected the physical interaction between these proteins using a co‐immunoprecipitation (CO‐IP) assay, which confirmed their physical interaction (Figure 2A). Protein–protein docking simulations supported potential protein interaction sites, showing a binding energy of −74.8905334. This robust interaction relied on hydrogen bonds and hydrophobic interactions, with a salt bridge and over 10 hydrogen bonds identified between Rnf40 and Parkin (Figure 2B). Immunofluorescence co‐localization analysis further demonstrated the co‐localization of Rnf40 and Parkin (Figure 2C). Using an MST assay, we demonstrated that Rnf40 directly interacts with Parkin in vitro (Figure 2D). Additionally, an in vivo ubiquitination assay revealed that Rnf40 induces Parkin ubiquitination (Figure 2E). Seven internal lysine residues (Lys6, 11, 27, 29, 33, 48, and 63) within ubiquitin can act as chain elongation sites, leading to various cellular processes, including degradation via the proteasome pathway [34]. In our study, we observed that Rnf40 promotes K48‐linked, but not K63‐linked, polyubiquitination of Parkin (Figure 2F).

**FIGURE 3:**
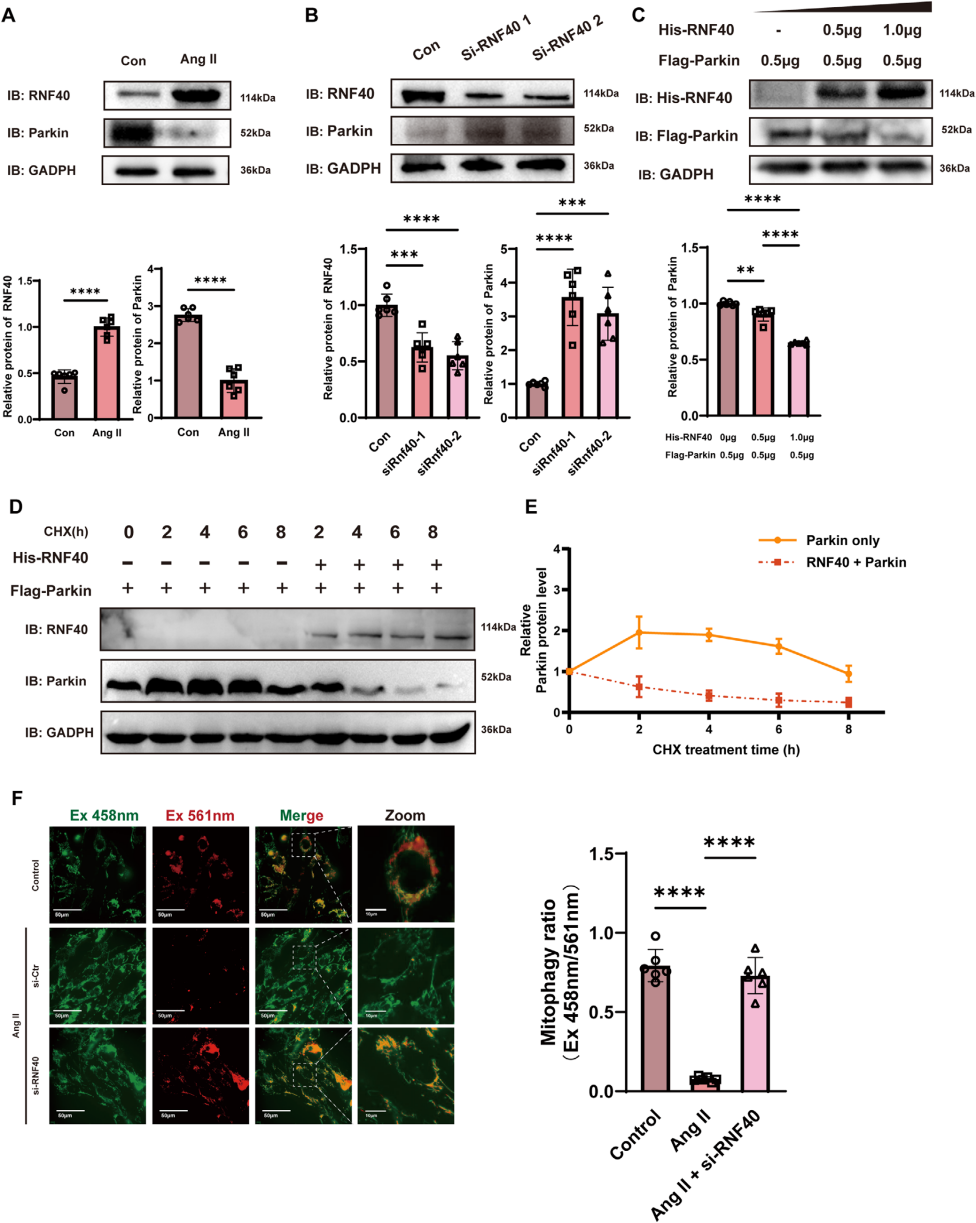
Rnf40 degrades Parkin and diminishes mitophagy. (A) Protein levels of Rnf40 and Parkin following Ang II treatment (100 nM) for 24 h in cerebrovascular tissue detected via Western blotting (mean ± SD, *n* = 6). (B) Western blot detection of Rnf40 and Parkin protein levels following Rnf40 knockdown (mean ± SD, *n* = 6). (C) Analysis of Parkin degradation in hCMEC/D3 cells co‐transfected with varying His‐Rnf40 amounts (mean ± SD, *n* = 6). (D and E) Determination of Parkin protein half‐life via Western blotting after treating hCMEC/D3 cells with cycloheximide (CHX, 100 mg/mL) (mean ± SD, *n* = 6). (D) Representative Western blot image. (E) Quantitative analysis of Parkin protein levels. (F) Representative image of mitophagy ratio in endothelial cells measured using Mt‐Keima staining. Scale bar = 50 μm. Mt‐Keima in free mitochondria has its excitation maxima near 458 nm at pH = 8, and mito‐lysosomes have an excitation maxima near 561 nm at pH = 4. The histogram depicts the ratiometric fluorescence for endothelial cells to describe the mitophagy ratio. One‐way ANOVA followed by the Tukey multiple comparison test was used to compare the difference between groups (mean ± SD, *n* = 6). Ang II, angiotensin II. **: *p* < 0.01; ***: *p* < 0.001; ****: *p* < 0.0001.

We next investigated whether Rnf40 degrades Parkin. hCMEC/D3 cells were treated with Ang II (100 nM) for 24 h, resulting in a significant increase in Rnf40 and a reduction in Parkin (Figure 3A). After knocking down Rnf40 in hCMEC/D3, we observed the upregulation of Parkin (Figure 3B). Overexpressing Rnf40 in hCMEC/D3 cells led to a dose‐dependent reduction in Parkin levels (Figure 3C). Protein stability assays using cycloheximide treatment and protein overexpression showed that Rnf40 mediates Parkin degradation (Figure 3D,E). Collectively, these results suggest that Rnf40, as an E3 ligase, could induce K48‐linked polyubiquitination and degradation of Parkin.

### 3.4 Rnf40 Reduces Mitophagy and Increases Endothelial Cell Permeability

**FIGURE 4:**
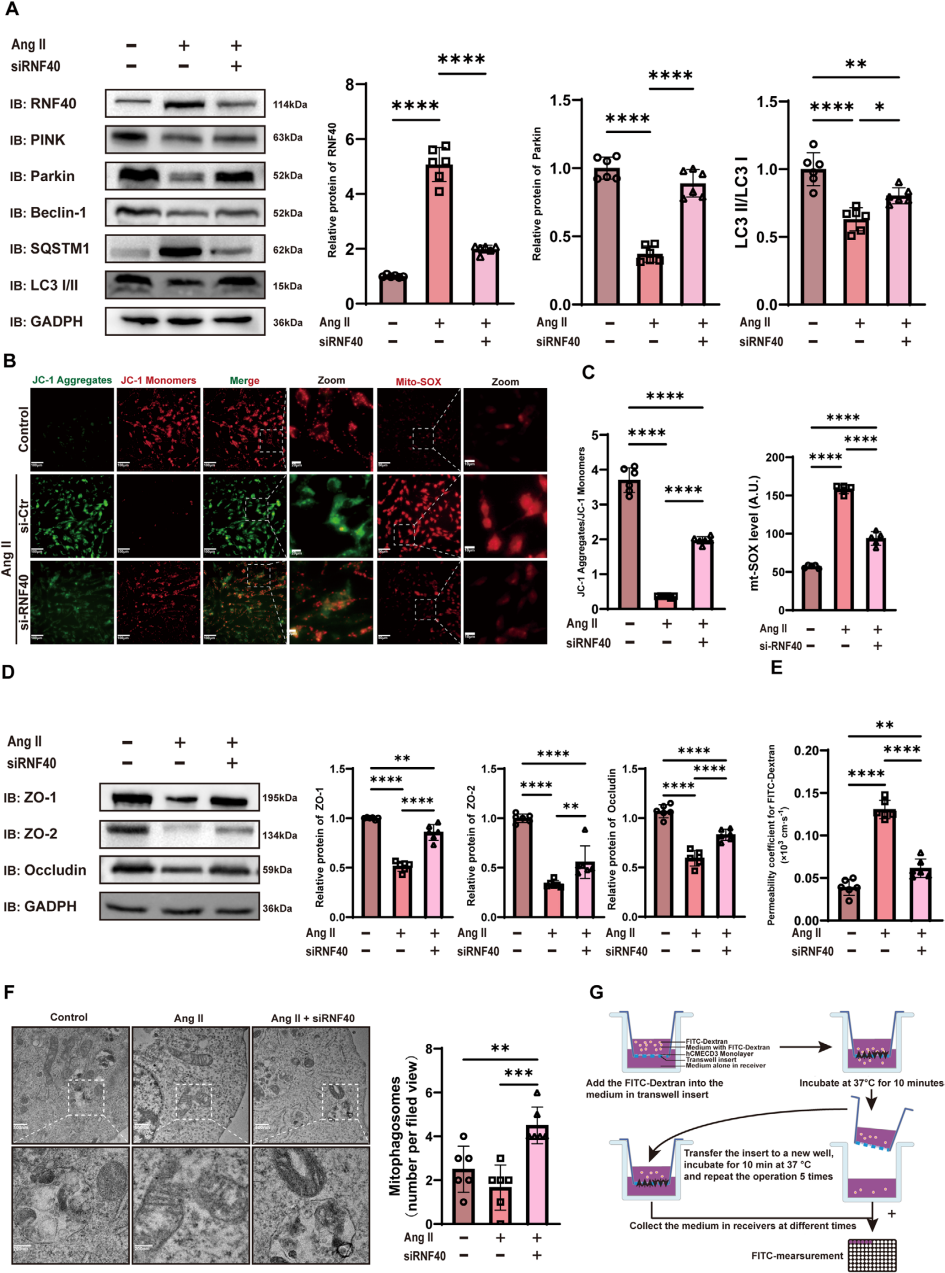
Rnf40 reduces mitophagy and increases endothelial cell permeability. (A) Protein levels of mitophagy markers following Ang II treatment (100 nM) for 24 h and Rnf40 knockdown in endothelial cells detected via Western blotting (mean ± SD, *n* = 6). (B and C) JC‐1 and mito‐SOX analysis in endothelial cells. (B) Representative images of JC‐1 and Mito‐SOX fluorescence microscopy. JC‐1 was used to access the mitochondrial membrane potential, and Mito‐SOX was used to quantify the mitochondrial superoxide levels. (C) Quantitative analysis of JC‐1 aggregate/monomer ratios and Mito‐SOX fluorescence intensities (mean ± SD, *n* = 6). (D) Protein levels of tight junction proteins detected using Western blotting after AngII treatment (100 nM) for 24 h and Rnf40 knockdown (mean ± SD, *n* = 6). (E) FITC–dextran permeability assay results depicting endothelial permeability. One‐way ANOVA followed by the Tukey multiple comparison test was used to compare the difference between groups (mean ± SD, *n* = 6). (F) Representative images of the endothelial cell mitochondrial structure using TEM with quantification of mitophagosomes (mean ± SD, *n* = 6). (G) The flow diagram of in vitro endothelial permeability assays. Ang II, angiotensin II; mito‐SOX, mitochondrial superoxide; FITC, fluorescein isothiocyanate; TEM: transmission electron microscopy. *: *p* < 0.05; **: *p* < 0.01; ***: *p* < 0.001; ****: *p* < 0.0001.

We explored the regulatory role of Rnf40 on mitophagy using Mt‐Keima, a probe that differentiates between mitochondria in the cytosol and those within autophagosomes/lysosomes. Small interfering ribonucleic acid (siRNA) targeting Rnf40 was used to knock down its expression. Our data showed that Ang II treatment impaired mitophagy in hCMEC/D3 cells, whereas Rnf40 knockdown restored it (Figure 3F). Transmission electron microscopy (TEM) revealed that in the Ang II intervention group, mitochondria displayed varying degrees of damage, including the loss of cristae integrity, swelling, and vacuolization. Despite these changes, the level of mitophagy remained unchanged. However, following the Rnf40 gene knockout, mitophagy showed partial recovery, and the extent of mitochondrial damage was reduced (Figure 4F). Western blot analysis indicated that Rnf40 mediates Ang II‐induced mitophagy deficiency (Figure 4A). Measurement of mitochondrial membrane potential and mitochondrial superoxide (mt‐SOX) confirmed that Ang II reduced membrane potential and elevated mt‐SOX release (Figure 4B,C). These findings highlight Rnf40 as a key mitophagy regulator.

Western blot analysis of tight junction proteins further indicated that Ang II treatment decreased tight junction protein expression, which was restored by Rnf40 silencing (Figure 4D). Subsequently, an FITC–dextran permeability assay demonstrated that Ang II increased endothelial permeability, and it could be attenuated via Rnf40 knockdown (Figure 4E). The findings show that Rnf40 silencing protects Ang II‐exposed cerebrovascular endothelial cells.

### 3.5 Rnf40 Increases Endothelial Permeability Through Modulation of Parkin‐Dependent Mitophagy

**FIGURE 5:**
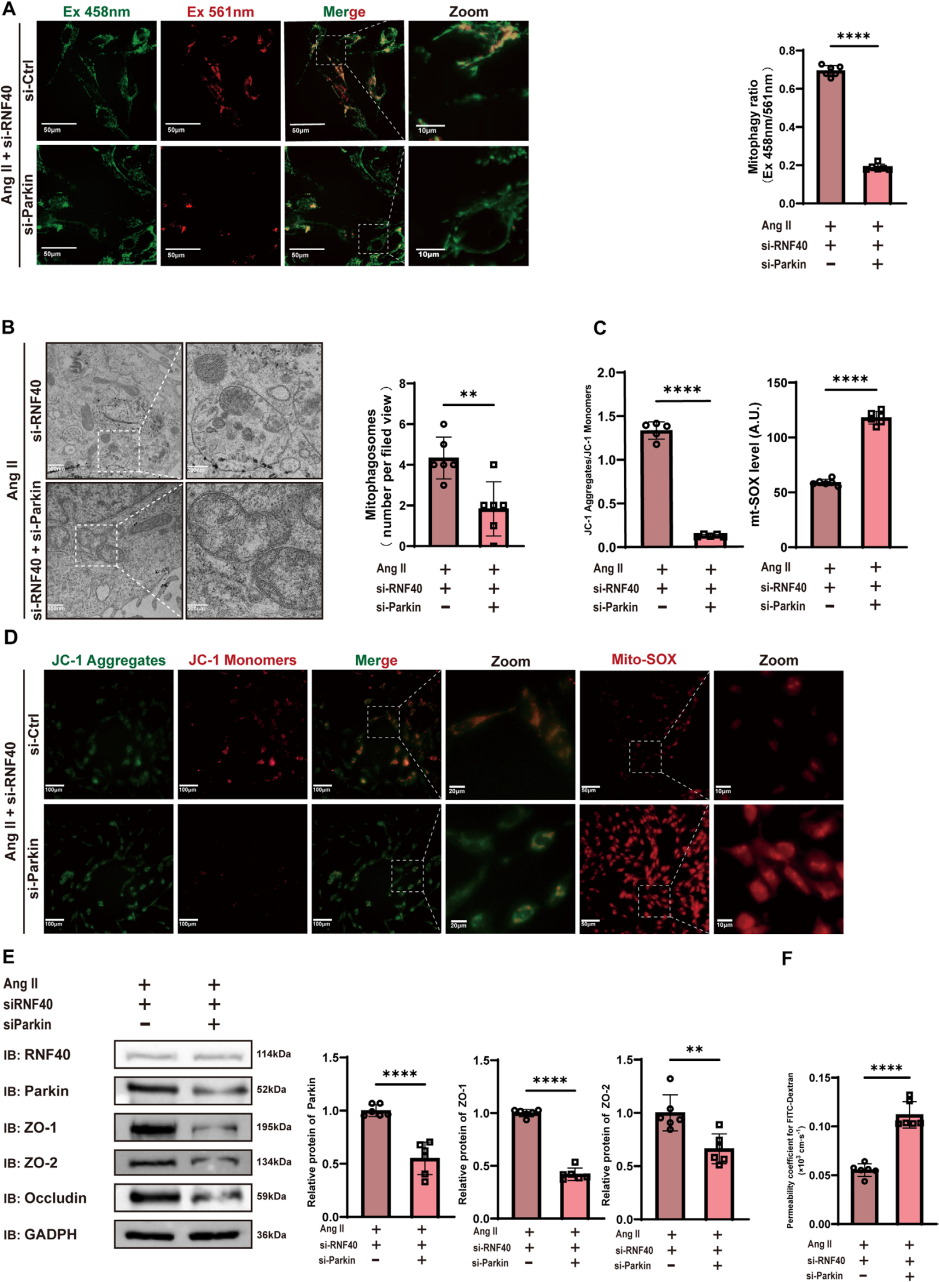
Rnf40 inhibits Parkin‐dependent mitophagy, increasing endothelial permeability. (A) Mitophagy ratio in endothelial cells assessed using Mt‐Keima staining after Parkin knockdown. Scale bar = 50 μm. The histogram depicts the ratiometric fluorescence for endothelial cells to describe the mitophagy ratio. Statistical significance was assessed using Student's *t*‐test (mean ± SD, *n* = 6). (B) Representative TEM images of the endothelial cell mitochondrial structure with quantification of mitophagosomes (mean ± SD, *n* = 6). (C and D) JC‐1 aggregate/monomer ratios and Mito‐SOX fluorescence analysis (mean ± SD, *n* = 6). (E) Protein levels of tight junction proteins following Parkin knockdown detected using Western blotting (mean ± SD, *n* = 6). (F) FITC–dextran permeability assay results depicting endothelial permeability after Parkin knockdown. One‐way ANOVA followed by the Tukey multiple comparison test was used to compare the difference between groups (mean ± SD, *n* = 6). Ang II, angiotensin II; mito‐SOX, mitochondrial superoxide; FITC, fluorescein isothiocyanate; TEM: transmission electron microscopy. **: *p* < 0.01; ****: *p* < 0.0001.

We examined the role of Parkin‐dependent mitophagy in Rnf40‐induced endothelial permeability. Using Ang II and Rnf40 siRNA treatment as the foundation, additional siRNA targeting Parkin was introduced to downregulate its expression. Parkin silencing inhibited mitophagy previously activated by Rnf40 knockdown (Figure 5A). The TEM revealed that that co‐knockdown of Parkin in endothelial cells, compared to that of RNF40 knockdown alone, led to pronounced mitochondrial damage, including compromised cristae integrity, mitochondrial swelling, and vacuolization, and a diminished level of mitophagy (Figure 5B). Furthermore, Parkin knockdown impaired the restorative effect of Rnf40 silencing on the mitochondrial membrane potential while also increasing mt‐SOX release (Figure 5C,D). Consistent with reduced mitophagy, Parkin knockdown diminished the expression of tight junction proteins (Figure 5E), thereby resulting in increased endothelial permeability (Figure 5F). Collectively, these results highlight that Rnf40 impairs the endothelial barrier function by degrading Parkin and modulating mitophagy.

### 3.6 Rnf40 Silencing Protects the Cognitive Function of SHRs Mainly Through Modulation of Parkin‐Dependent Mitophagy

To elucidate the role of RNF40 in hypertension‐induced cerebrovascular endothelial cell damage, we established an Rnf40‐depleted model in SHRs. Moreover, TRV027, a β‐arrestin‐biased angiotensin type 1 receptor agonist, was administered to SHRs as a positive control to reduce blood pressure.

**FIGURE 6:**
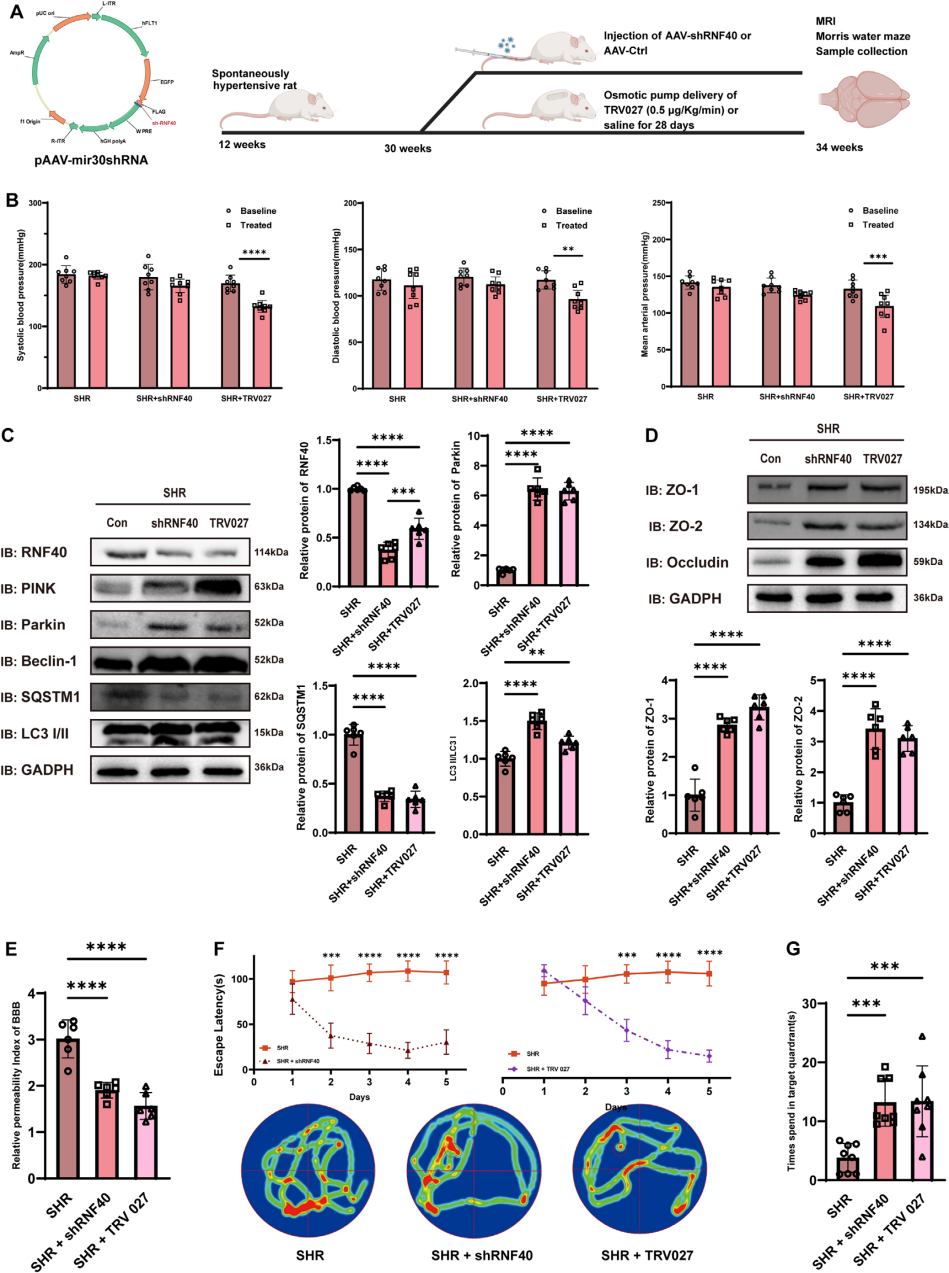
Silencing Rnf40 modulates Parkin‐dependent mitophagy and preserves cognitive function in SHRs. (A) AAV vector structure and experimental flow diagram (Created in BioRender. Kou, C. (2024) BioRender.com/c52o084). (B) The blood pressure after treatment for SHRs (mean ± SD, *n* = 8). (C) Western blot analysis of Rnf40 and mitophagy protein levels following Rnf40 knockdown (mean ± SD, *n* = 6). (D) Tight junction protein levels detected using Western blotting (mean ± SD, *n* = 6). (E) BBB permeability was assessed using the FITC–dextran assay. One‐way ANOVA with Tukey's test was used to compare the difference between groups (mean ± SD, *n* = 6). (F) Escape latency and heatmap analysis in the MWM. Statistical significance was assessed using Student's *t*‐test (mean ± SD, *n* = 8). (G) Time spent in the quadrant area of the MWM probe trial. One‐way ANOVA followed by the Tukey multiple comparison test was used to compare the difference between groups (mean ± SD, *n* = 8). BBB, blood brain barrier; MWM, morris water maze; SHRs, spontaneously hypertensive rats. **: *p* < 0.01; ***: *p* < 0.001; ****: *p* < 0.0001.

After treatment, we observed that TRV027 significantly reduced blood pressure in SHRs, whereas the knockdown of Rnf40 did not significantly affect blood pressure in SHRs (Figure 6B). We then assessed mitophagy protein levels via Western blot analysis. Consistent with in vitro trials, both TRV027 treatment and Rnf40 silencing restored Parkin expression, significantly increasing the levels of mitophagy proteins (Figure 6C). Similarly, the relative level of tight junction proteins improved following Rnf40 knockdown (Figure 6D). Data from the in vivo FITC–dextran permeability assay confirmed that Rnf40 knockdown ameliorated the cerebrovascular endothelial barrier dysfunction (Figure 6E).

The MWM was performed to evaluate global cognitive function. Rnf40 silencing reduced escape latency significantly in SHRs from training days 2–5 (Figure 6F) and increased time spent in target quadrants (Figure 6G). The results indicated that Rnf40 silencing effectively mitigated hypertension‐induced cognitive dysfunction. Furthermore, brain structures and rCBF were assessed using MRI. No significant difference was observed in cortex or hippocampus volumes (Figure S3B,C), but ASL data showed that rCBF was significantly reduced in the cortex and hippocampus of SHRs. Rnf40 knockdown restored the reduced rCBF (Figure S3D,E).

## 4 Discussion

Cerebrovascular damage significantly contributes to hypertension‐induced cognitive impairment. In this study, we demonstrated that mitophagy deficiency is a critical mechanism favoring hypertension‐induced cerebrovascular endothelial barrier dysfunction. We further discovered that Rnf40, as an E3 ubiquitin ligase for Parkin, promotes K48‐linked polyubiquitination and degradation of Parkin. Rnf40 silencing inhibited Parkin degradation and restored mitophagy in cerebrovascular cells, thus preserving endothelial barrier function and cognitive abilities. Collectively, our findings provide evidence that Rnf40 directly inhibits mitophagy by ubiquitinating and degrading Parkin, a key mechanism underlying hypertension‐induced dysfunction in cerebrovascular endothelial barriers.

Hypertension is a major risk factor for cardiovascular disease and mortality globally [35], causing systemic organ damage. In the present study, SHR was used as a model of hypertension to investigate the mechanisms underlying the impact of high blood pressure on cerebrovascular damage and cognitive impairment. Previous studies have demonstrated that SHR rats exhibit a significant increase in blood pressure by 12 weeks of age and develop localized BBB dysfunction, white matter injury, and microglial activation by 34–35 weeks, which qualifies them as a model for early‐stage CSVD [31]. Based on this, we selected 34‐week‐old SHR rats for the present study. Our findings revealed significant cognitive impairment in the rats at 34 weeks, along with endothelial dysfunction and reduced CBF, consistent with earlier research [31]. Mitophagy plays a pivotal role in hypertension‐mediated organ damage. Hypertension can reportedly result in either insufficient or excessive mitophagy, both of which adversely affect mitochondrial homeostasis. Typically, hypertension is associated with mitophagy deficiency [36]. However, the impact of hypertension on the mitophagy of cerebrovascular mitophagy remains limited. Reactivating autophagy and mitophagy could reportedly restore the cerebrovascular function in stroke‐prone spontaneously hypertensive rats (SHR/SP) [37]. Moreover, in a mouse model infused with Ang II, reduced cardiac vascular mitophagy correlated with microvascular rarefaction [38]. Consistently, in this study, we observed mitophagy deficiency in the cerebrovascular system of SHRs. Recent studies suggest that mitophagy activation can protect endothelial function and prevent BBB damage in traumatic brain injury [39]. Nevertheless, excessive mitophagy has been implicated in endothelial dysfunction and tight junction protein degradation due to methylglyoxal [40]. Thus, the relationship between mitophagy and cerebrovascular endothelial barrier function under hypertensive conditions remains unclear. Our data further revealed an association between mitophagy and cerebrovascular endothelial barrier function. Deficient mitophagy could cause endothelial barrier dysfunction, which adversely affects cognitive performance in SHRs. Modulating Parkin expression can effectively regulate mitophagy levels [16]; therefore, we controlled Parkin expression levels through post‐translational modifications to further modulate mitophagy in this study. Our data indicate that restoring Parkin expression effectively enhances mitophagy and improves cerebrovascular endothelial barrier function in SHRs.

We identified Rnf40 as a novel E3 ligase of Parkin. Rnf40 is composed of four coiled‐coil domains and a C‐terminal RING finger domain, with the latter playing a crucial role in both protein–DNA and protein–protein interactions [41]. This enzyme was initially identified in rats by Chin et al.; this enzyme targets Syntaxin‐1 for degradation via the ubiquitin–proteasome pathway [19, 42]. Furthermore, Rnf40 regulates histone H2B ubiquitination and other non‐histone proteins [20, 43, 44]. Current research on Rnf40 primarily focuses on its role in breast cancer. One study reported that the knockdown of the H2B ubiquitin ligase Rnf40 reduced ERα‐induced transcription and activated the signaling pathways associated with estrogen‐dependent cell proliferation and cell survival, thereby exerting a tumor‐suppressive role in breast cancer [45]. Meanwhile, Rnf40 also plays a crucial role in conditions such as inflammatory bowel disease [20], type‐1 diabetes [46], and cancer‐associated osteolysis [23].

Through DNA microarrays, UbiBrowser 2.0, and MS, we identified Rnf40 as a potential E3 ligase for Parkin. CO‐IP was used to confirm their interaction in vivo, while MST validated their direct interaction in vitro. In the PINK–Parkin axis, PINK accumulates on defective mitochondria and triggers the translocation of Parkin from the cytosol [47], thereby promoting mitophagy. Post‐translational modifications, such as phosphorylation and ubiquitination, are crucial to this process [48, 49], Notably, direct evidence identifying a specific E3 ubiquitin ligase responsible for ubiquitinating Parkin is lacking. Though most studies focus on Parkin substrates and deubiquitinating enzymes (DUBs), no E3 ubiquitin ligase has been identified [50], Notably, DUBs affect Parkin via varied deubiquitination processes, leading to distinct mitophagy outcomes. Therefore, these proteins can serve as regulators in mitophagy. Among these proteins, ubiquitin‐specific peptidase 33 (USP33) removes K63‐linked ubiquitin chains, destabilizing Parkin and suppressing mitophagy [33], Conversely, USP8 is capable of removing the K6‐linked ubiquitination from Parkin, thus stabilizing the protein and promoting mitophagy [51], Thus, targeting distinct Parkin ubiquitination modifications yields different outcomes. In our in vivo ubiquitination assay, Rnf40 facilitated K48‐linked polyubiquitination, thus promoting Parkin degradation via the ubiquitin–proteasome pathway.

We acknowledge several limitations in our study. First, due to the challenge of obtaining cerebrovascular samples from patients with hypertension, our findings could not be validated in clinical samples. The direct applicability of our findings from animal models to humans remains limited owing to differences between species. Additionally, more specific techniques are required to precisely reduce Rnf40 expression in cerebrovascular endothelial cells in vivo to further clarify its role in hypertension‐induced cerebrovascular endothelial barrier dysfunction. Finally, these findings require validation in large‐scale clinical trials.

## 5 Conclusion

We identified a novel E3 ligase, Rnf40, which is overexpressed in SHRs and facilitates K48‐linked polyubiquitination and degradation of Parkin. This action inhibits mitophagy in cerebrovascular endothelial cells, leading to endothelial barrier dysfunction. Knocking down Rnf40 could effectively reverse this process, thereby improving hypertension‐induced cerebrovascular endothelial barrier dysfunction and cognitive performance.

## Author Contributions

Jing Yu conceptualized the study. Chengkun Kou and Xu Zhao conducted research, analyzed the data, and drafted the article. Xin Fan, Runmin Sun, Wenting Wang, Miaomiao Qi, Lulu Zhu, and Xin Lin provided materials and reviewed and edited the article. Jing Yu secured funding, interpreted the data, and edited the article.

## Disclosure

The authors have nothing to report.

## Conflicts of Interest

The authors declare no conflicts of interest.

## Supporting information


Appendix S1.

## Data Availability

All supporting data and study details are available within the article and Supporting Information. The original data generated in this study can be obtained from the corresponding author upon request.
