# Supplementary material for: Rnf40 Exacerbates Hypertension‐Induced Cerebrovascular Endothelial Barrier Dysfunction by Ubiquitination and Degradation of Parkin
Source: CNS Neurosci Ther. 2025 Jan 8;31(1):e70210. doi: 10.1111/cns.70210 (PMC11707429; doi:10.1111/cns.70210)
Supplement: Supplementary file 1 — Appendix S1. [file CNS-31-e70210-s001.docx]

**Supplemental Material**

Title: Rnf40 Exacerbates Hypertension-Induced Cerebrovascular Endothelial Barrier Dysfunction by Ubiquitination and Degradation of Parkin

**Authors:** Chengkun Kou^1,2,†^, Xu Zhao^1,2,†^, Xin Fan^1,2^, Runmin Sun^1,2^, Wenting Wang^1,2^, Miaomiao Qi^1,2^, Lulu Zhu^1,2^, Xin Lin^1^, Jing Yu^1,2,3,*^

**Affiliations:**

^1^ Hypertension Center, Lanzhou University Second Hospital, Lanzhou, Gansu, China.

^2^ The Second Clinical Medical School, Lanzhou University, Lanzhou, Gansu, China.

^3^ Cuiying Biomedical Research Center, Lanzhou University Second Hospital, Lanzhou, Gansu, China.

^†^ These authors contributed equally to this study.

^*^ Corresponding Author: Jing Yu. Email: ery_jyu@lzu.edu.cn. Address: No. 82 Cuiyingmen Lanzhou, Gansu province, P R. China. Zip code: 730030

**Materials and Methods**

**Immunoblotting**

Tissues or cells were lysed using 60ul RIPA buffer (tissues: 20mg/150ul, cells: 2×10^6^/ul) in ice for 20 min. And then centrifuged at 12000rpm for 15 min to remove the debris and nuclei. And boiled for 5 minutes with SDS-buffer in 95°C. Finally, samples were used to perform western blot assay. The information of antibody used in this study were listed in Table S1.

**Isolation of cerebrovascular**

Removing the cerebellum and olfactory bulb initially. Subsequently, the remaining brain tissue was immediately preserved by rapid freezing in liquid nitrogen and maintained at −80°C for future analysis. For homogenization, the tissue was processed in a Dounce homogenizer using a tight-fitting pestle with 3 ml of cold 0.32 M sucrose buffer (5 mM HEPES, pH 7.4). The homogenate underwent centrifugation at 1000g for 10 minutes, post which the supernatant predominantly containing neuronal components was discarded. Additionally, the superficial myelin layer was carefully removed from the pellet and discarded. The pellet was resuspended in the same sucrose buffer and re-centrifuged under identical conditions to ensure complete myelin removal. For further purification, the sediment was subjected to a centrifugation at 40g for 10min to segregate large blood vessels from capillaries. The final pellet underwent four additional wash cycles with 1 ml of sucrose buffer, each followed by centrifugation at 350g for 10 minutes.

**RT-PCR**

Total RNA was isolated from the cerebrovascular tissues of SHR rats using TRIzol reagent (Invitrogen). This was followed by reverse transcription using a kit (RR037A, Takara Bio, Japan). Quantitative real-time PCR was then performed to assess the mRNA expression levels of Rnf40 using a LightCycler system (Takara Bio, Japan) and iTaq Universal TB Green Supermix (Takara Bio, Japan). The specific primer sequences for Rnf40 and β-actin are detailed in Table S2. Expression data were analyzed using the ΔΔCT method to calculate relative changes in gene expression.

**Immunohistochemistry**

Rat tissues were fixed in formalin and embedded in paraffin. The sections underwent deparaffinization, rehydration, antigen retrieval, and blocking of endogenous peroxidases. Subsequently, they were washed three times in 0.01 mol/L PBS for 5 minutes each. Sections were then blocked for 1 hour in 0.01 mol/L PBS enhanced with 0.3% Triton X-100 and 5% normal goat serum. This was followed by overnight incubation at 4°C with primary antibodies against Rnf40 (1:100). After rinsing briefly in 0.01 mol/L PBS, the sections were incubated for 2 hours with horseradish peroxidase–conjugated rabbit anti-goat immunoglobulin G (1:500) in 0.01 mol/L PBS. Development was achieved using 0.003% H_2_O_2_ and 0.03% 3,3’-diaminobenzidine in 0.05mol/L Tris-HCl (pH 7.6). Immunohistochemical analysis was performed at least three times for each sample, and sections were counterstained with hematoxylin to enhance visualization.

**Immunofluorescence**

Cells were fixed in methanol at room temperature for 30 minutes and subsequently blocked with 2% bovine serum albumin for 1 hour at room temperature. Primary antibodies (Rnf40 at 1:100, Parkin at 1:50) were applied and incubated overnight at 4°C. Following extensive washing with phosphate-buffered saline (PBS), secondary antibody incubation (1:200) was conducted at room temperature for 1 hour. The samples were then washed again with PBS. Finally, DAPI staining was performed at a dilution of 1:200 for 15 minutes at room temperature.

**Cell Transfection**

For siRNA transfection, the hCMEC/d3 cell line was seeded at a density of 1 × 10^5 cells per well in 6-well plates. Transfection was carried out using Lipo8000 reagent, following the manufacturer’s instructions (C0533, Beyotime Biotechnology, China). Specifically, cells were transfected with siRnf40 (stB0009183A-1-5 & stB0009183B-1-5, RiboBio Co., Ltd., China), siParkin (siG1172283919-1-5, RiboBio Co., Ltd., China), or a negative control siRNA (siN0000001-1-10, RiboBio Co., Ltd., China). The efficacy of silencing was assessed by Western blot analysis.

For plasmid transfection, HEK-293T and hCMEC/d3 cells were plated in 6-well plates. After 16 hours, plasmids (1µg) were introduced using Lipo8000 reagent, following the protocol provided by the manufacturer (C0533, Beyotime Biotechnology, China).

**Virus Production and Infection**

HEK-293T cells (1×10^6^) were seeded in 6-well plates and allowed to adhere for 16 hours. Subsequently, they were transfected with pLV3-CMV-mito-mKeima-Puro along with lentiviral packaging plasmids psPAX2 and pMD2.G in opti-MEM medium using Lipo8000 (Beyotime Biotechnology, China) Following a 6-hour incubation, the transfection medium was replaced with DMEM supplemented with 10% fetal bovine serum. After 36 hours post-transfection, centrifugation was performed to remove cell debris, followed by the addition of 1 ml lentivirus and 2 μl polybrene to infect the cells.

**Magnetic Resonance Imaging**

Animals were anesthetized with isoflurane anesthesia (4% for induction and 2% for maintenance) in a mixture of O2 and air (30/70%). Subsequently, the animals were prepared for mechanical ventilation by endotracheal intubation. Animals were immobilized in a specially designed stereotactic holder and cradle to minimize movement during the MRI experiment. For volumetric analyses and registration, T1-weighted images were performed. Field-of-view (FOV) =0.20× 0.19 × 0.19 mm3; acquisition matrix = 92 × 512 × 512 points. Isoflurane anesthesia level was reduced to 1.5% at the start of the anatomical MRI acquisition, to lower the anesthetic depth for the following resting-state fMRI acquisition. Arterial spin-labeling (ASL) perfusion maps were obtained on a single axial slice of the brain located on the point of the rostro–caudal axis where the hippocampus had the largest cross section. The imaging geometry was a 0.625 × 0.625 mm2 field-of-view (FOV) of 2 mm in thickness, with a single-shot echo-planar encoding over a 128 × 128 matrix.

**Table S1.** The information of antibody in this study.

| Antibodies | SOURCE | IDENTIFIER |
| --- | --- | --- |
| Rnf40 | Abcam | ab191309 |
| Parkin | Abcam | ab77924 |
| HA | Proteintech | 81290-1-RR |
| Flag | Proteintech | 66008-4-Ig |
| His | Proteintech | 66005-1-Ig |
| ZO-1 | Abcam | ab190085 |
| ZO-2 | CST | #2847 |
| Occludin | Proteintech | 27260-1-AP |
| PINK | Proteintech | 23274-1-AP |
| Beclin-1 | Proteintech | 11306-1-AP |
| SQSTM1 | Proteintech | 18420-1-AP |
| LC3 I/II | Proteintech | 14600-1-AP |
| CD31 | Proteintech | 11265-1-AP |
| eNOS | Proteintech | 27120-1-AP |
| GAPDH | Proteintech | 60004-1-Ig |

**Table S2.** The information of Primer in this study.

| **Name** | **Primer sequence（5'-3')** |
| --- | --- |
| **Rnf40-F** | TGCTACGAGAACCAGAGGGA |
| **Rnf40-R** | CCGGCTGTATACTCGCTGAC |
| **β-actin-F** | AGATCCTGACCGAGCGTGGC |
| **β-actin-R** | CCAGGGAGGAAGAGGATGCG |


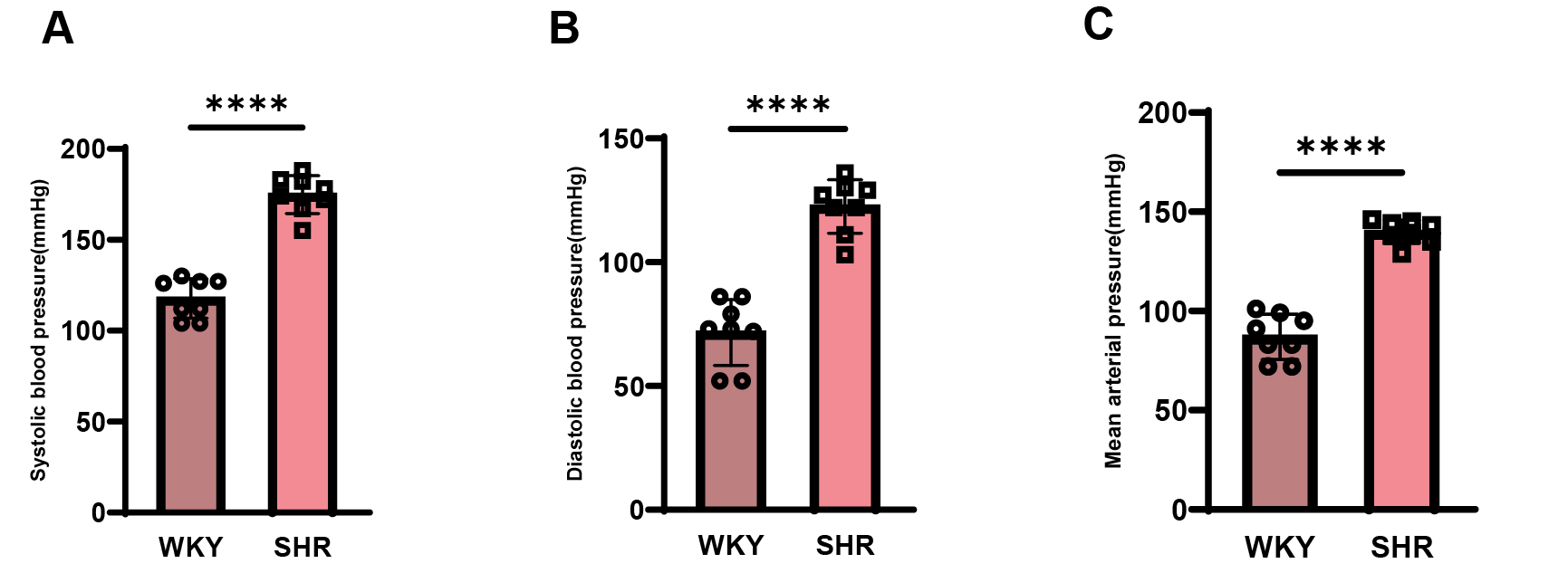


**Figure S1.** A, Systolic blood pressure at baseline (n = 8). B, Diastolic blood pressure at baseline (n = 8). C, Mean arterial pressure at baseline (n = 8).


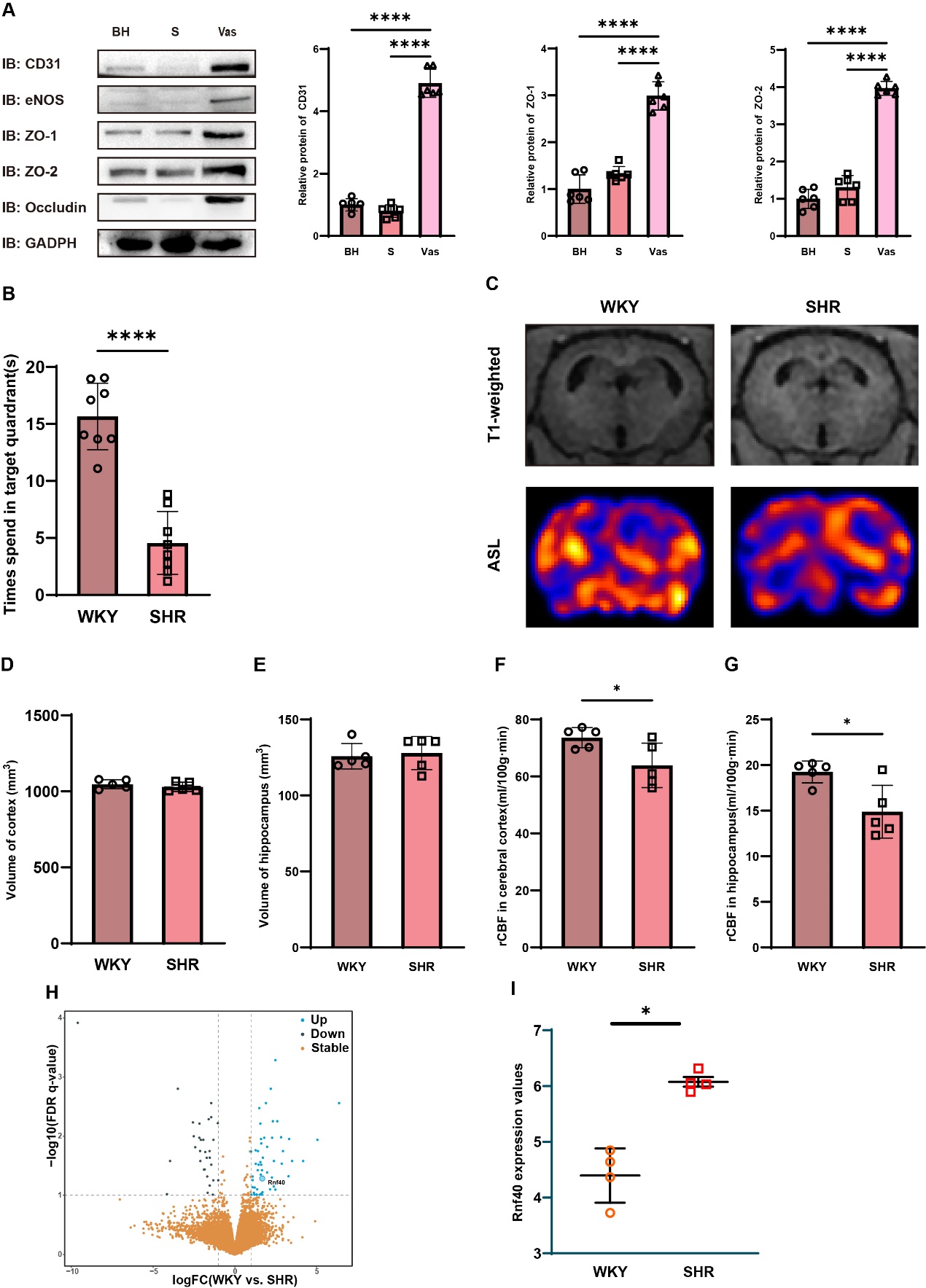


**Figure S2.** A, The protein level of endothelial biomarker in vasculature isolated (mean ± S.D., n=6). B, Time spent in the quadrant area in the probe trial of MWM, Student-t test was used to compare the difference between groups (mean ± S.D., n=8). C, Representative image of T1-weighted and ASL for WKYs and SHRs. D, Comparation of cortex volume between WKYs and SHRs. E, Comparation of hippocampus volume between WKYs and SHRs. F, Comparation of cortex rCBF between WKYs and SHRs. G, Comparation of hippocampus rCBF between WKYs and SHRs. H, Volcano plot displayed 53 genes to be up-regulated in SHRs and 31 genes down-regulated s(*P*<0.05). H, The mRNA expression level of Rnf40 in GSE74288.


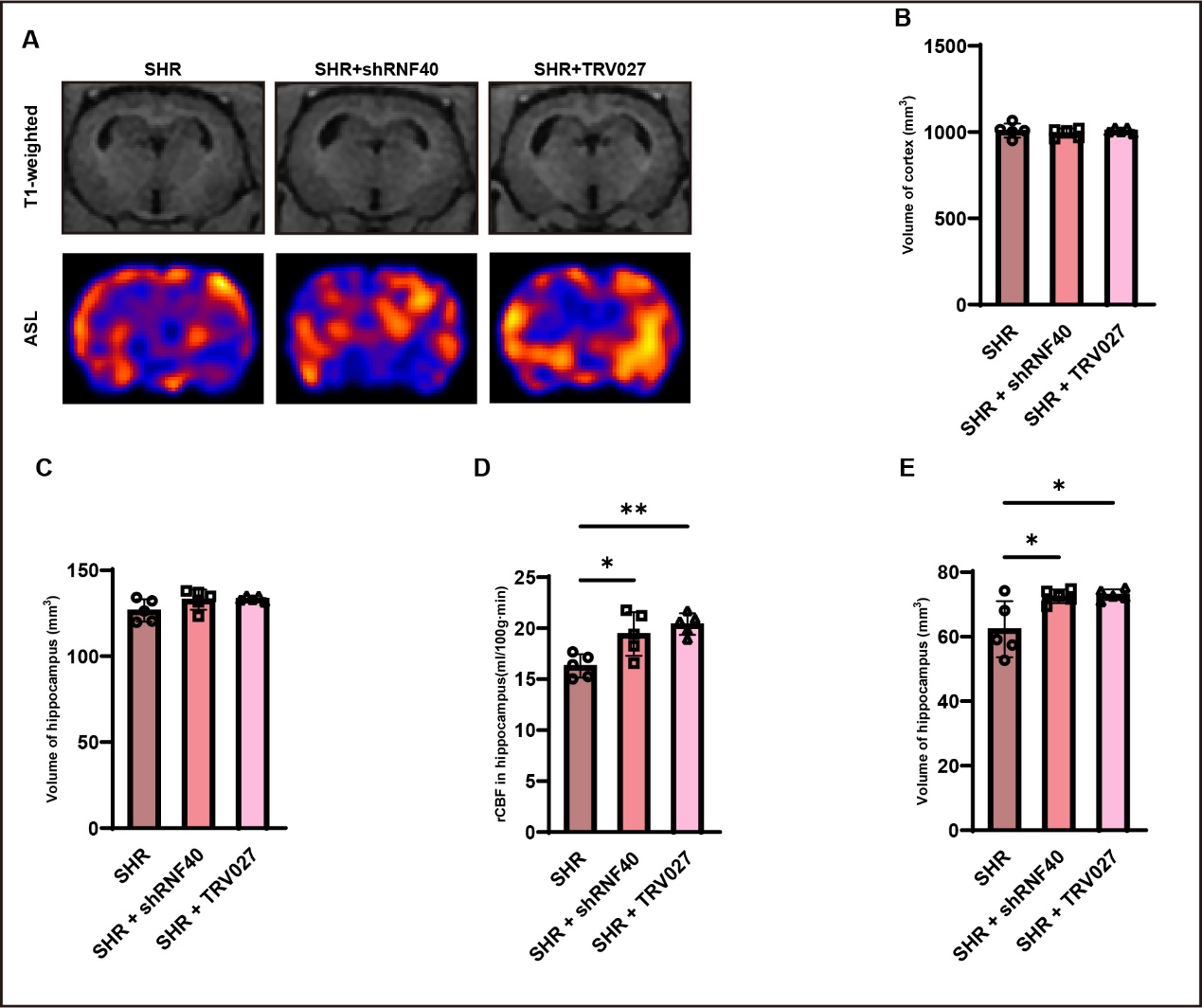


**Figure S3.** A, Representative image of T1-weighted and ASL of SHRs after treatment. B, Comparation of cortex volume between WKYs and SHRs. E, Comparation of hippocampus volume between WKYs and SHRs. F, Comparation of cortex rCBF between WKYs and SHRs. One-way ANOVA followed by Tukey multiple comparisons test were used to compare the difference between groups (mean ± S.D., n=8)
